# Dissecting Metabolic Rewiring and Gene-Metabolite Interactions by Utilizing Untargeted Metabolomics and Single-Gene Knockouts in the Model Microorganism *E. coli*

**DOI:** 10.1021/jasms.5c00454

**Published:** 2026-03-16

**Authors:** Xinru Pang, Li Chen, Huan Zhang, Shiqi Zhang, Jiangjiang Zhu

**Affiliations:** Department of Human Sciences & James Comprehensive Cancer Center, 2647 The Ohio State University, Columbus, Ohio 43210, United States of America

**Keywords:** untargeted metabolomics, single-gene knockout model, *Escherichia coli*, metabolic rewiring, gene-metabolite interaction, central carbon metabolism

## Abstract

Central carbon metabolism, comprising glycolysis, the tricarboxylic acid (TCA) cycle, and the pentose phosphate pathway (PPP), is essential for *Escherichia coli* survival and growth. While disruptions in these pathways are known to affect cellular physiology, the system-wide metabolite-level consequences of single-gene knockouts remain incompletely understood. Using untargeted LC-MS metabolomics, we systematically profiled *E. coli* knockouts of TCA core enzymes, isoforms, subunits, bypass routes, and TCA-associated pathways. Core TCA knockouts separated into two major metabolic clusters, with cluster 1 strains displaying strong divergence in amino acid metabolism and cluster 2 retaining partial similarity to the parent strain. Isoform-specific deletions revealed differential roles of aconitases (Δ*acnA* vs Δ*acnB*) and fumarases (Δ*fumA* vs Δ*fumC*), while subunit knockouts of 2-oxoglutarate dehydrogenase (Δ*sucA*, Δ*sucB*) and succinate dehydrogenase (Δ*sdhA-D*) produced localized but distinct metabolite shifts, particularly around glutamate- and 2-oxoglutarate-linked metabolism. Bypass enzyme deletions (Δ*aceA*, Δ*aceB*, Δ*glcB*, and Δ*maeB*) disrupted carbohydrate- and redox-related metabolites, underscoring their role as metabolic safety nets. Importantly, knockouts also triggered off-target effects in glycolysis, PPP, and the electron transport chain, highlighting the interconnectivity of central carbon metabolism. Our systematic approach demonstrated the possibility of utilizing comprehensive and untargeted metabolomics to map gene-metabolite associations and decipher potential metabolic interlinks.

## Graphical abstract

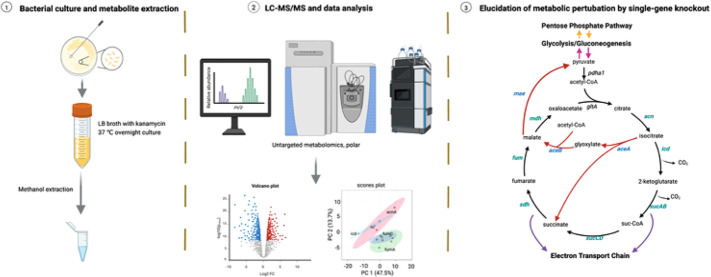

## 1 Introduction

Major metabolic pathways, such as the central carbon metabolism in *Escherichia coli* (*E. coli*), are highly robust, integrating the tricarboxylic acid cycle (TCA), glycolysis, gluconeogenesis, pentose phosphate pathway (PPP), and the electron transport chain (ETC) to support energy production, biosynthesis, and redox balance.^1,2^ The TCA cycle is a central hub in this network, generating reducing equivalents for oxidative phosphorylation while supplying precursors for amino acid, nucleotide, and cofactor biosynthesis.^3^ Importantly, the cycle is not a closed loop but is complemented by bypass routes, such as the glyoxylate shunt and malic enzyme-mediated bypass, which provide metabolic flexibility under stress or genetic perturbations.^4,5^

Single-gene knockouts have been instrumental in probing the metabolic function and plasticity in microbes. Studies using the Keio Collection, a systematic library of *E. coli* single-gene deletions, have revealed that even nonessential gene knockouts can induce global metabolic reprogramming and trigger compensatory fluxes across central metabolism.^6^ In many cases, isoform- or subunit-specific gene deletions within multienzyme complexes result in nuanced or distinct phenotypes, reflecting partial redundancy and distributed control of flux.^7,8^ For example, *E. coli* encodes multiple isoforms of aconitase (AcnA/B) and fumarase (FumA/B/C) that contribute differentially to TCA activity depending on environmental and cellular states.

While the direct roles of TCA cycle enzymes are well characterized, the broader metabolite-level consequences of their knockout remain underexplored. Knockouts can propagate off-target effects across interconnected pathways, such as PPP, glycolysis, and ETC, driven by metabolite accumulation, redox imbalance, or global regulatory responses.^6^ These secondary effects are difficult to predict, yet they provide critical insight into the plasticity and adaptability of bacterial metabolism. Untargeted metabolomics offers a powerful approach to capture these system-wide metabolic shifts, enabling the discovery of hidden phenotypes and providing mechanistic insights into metabolic resilience.

Here, we use single-gene knockouts in *E. coli* as a model system to demonstrate how untargeted metabolomics can uncover unexpected and previously unreported metabolic changes arising from the deletion of enzymes involved in the TCA cycle and its associated pathways. By systematically profiling these knockout mutants, we reveal distinct metabolic rewiring patterns, unexpected metabolite shifts, and hidden network interconnections that extend beyond canonical pathway annotations. Beyond advancing the fundamental understanding of metabolic robustness, these findings have direct relevance for synthetic biology and metabolic engineering by identifying metabolic nodes that exhibit high buffering capacity versus those that induce widespread network reorganization when perturbed. Such gene-metabolite maps provide a data-driven framework for predicting off-target effects, optimizing flux redistribution, and improving robustness in engineered microbial systems.

## 2 Materials and Methods

### 2.1 Chemical Reagents and Bacterial Culture Supplies

LB (Lysogeny Broth) agar and LB broth medium used for culturing *E. coli* were purchased from Fisher Scientific (Hampton, NH, USA). Petri dishes, conical tubes, and sterile Eppendorf tubes used for culturing and metabolite extraction were also sourced from Fisher Scientific. Kanamycin for antibiotic selection was acquired from MilliporeSigma (Burlington, MA, USA).

Mobile phases for metabolomic analysis were prepared by using ammonium acetate (MilliporeSigma, Burlington, MA, USA), acetonitrile (Sigma-Aldrich, St. Louis, MO, USA), and acetic acid (Fisher Scientific, Hampton, NH, USA). Stable isotope-labeled amino acids, used as internal standards, were purchased from Cambridge Isotope Laboratories (Tewksbury, MA, USA).

### 2.2 *E. coli* Culturing in LB Broth with Kanamycin

Ninety*E. coli*Keio knockout strains and the corresponding wild-type parent strain were obtained from Horizon Discovery (Cambridge, United Kingdom). For culturing, LB broth was prepared using 20 g/L of low-salt LB medium dissolved in 920 mL of deionized water. Kanamycin was added to a final concentration of 25 μg/mL. The medium was sterilized by autoclaving at 121°C for 25 min.

Frozen stocks of*E. coli*strains were revived by streaking onto LB agar plates. After incubation, single colonies were isolated and cultured overnight in an LB broth. Four biological replicates were prepared in each experiment. A 100 μL aliquot of the overnight culture was then transferred to a 15 mL conical tube containing 10 mL of fresh LB broth supplemented with kanamycin. Cultures were incubated aerobically at 37 °C for 24 h.

After incubation and the OD test, 1 mL of the bacterial suspension was transferred to sterile Eppendorf tubes. The wild-type parent strain was cultured in LB broth without kanamycin and utilized as a control for baseline comparison. LB broth without bacterial inoculation was included as a negative control.

### 2.3 Metabolites Extraction from *E. coli* Cultures

For metabolite extraction, bacterial cultures stored in sterile microcentrifuge tubes were centrifuged at 14,000 rpm for 10 min at 4 °C using a Thermo Scientific Sorvall ST 8 Centrifuge. The bacterial pellets (intracellular fraction) were washed three times with 500 μL of DPBS. In each wash, the pellets were resuspended by pipetting up and down to ensure adequate contact with the buffer, followed by centrifugation at 14000 rpm for 10 min at 4 °C. Supernatants were discarded after each wash. Polar metabolites were extracted by adding 250 μL of methanol, followed by the addition of 50 μL of a stable isotope-labeled amino acid mixture, used as internal standards. The mixtures were vortexed for 2 min. Samples were then placed at −20 °C for 20 min to enhance metabolite precipitation. After cooling, the mixtures were centrifuged again at 14,000 rpm for 20 min at 4 °C. A 150 μL aliquot of the supernatant was transferred to new sterile 1.5 mL microcentrifuge tubes and dried using a SpeedVac concentrator at 30 °C for approximately 1.5 h. The dried extracts were reconstituted in 200 μL of 50% acetonitrile (v/v, acetonitrile:deionized water = 1:1), vortexed for 2 min, and transferred into plastic inserts placed inside glass LC vials. A 10 μL aliquot from each sample was pooled into a separate glass vial to serve as a quality control (QC) sample for polar metabolite analysis.

### 2.4 Data Acquisition, Processing, and Statistical Analysis of Untargeted Metabolomics

The untargeted metabolomics workflow applied in this study was previously described in detail.^9^ Metabolites were analyzed using a Thermo Vanquish ultraperformance liquid chromatography (UPLC) system (Thermo Fisher Scientific, Waltham, MA, USA) coupled to a hybrid quadrupole-Orbitrap mass spectrometer (Q Exactive Plus, Thermo Fisher Scientific). Chromatographic separation was performed on an XBridge BEH Amide column (2.5 μm, 2.1 × 150 mm; Waters, Milford, MA, USA), optimized for polar metabolite retention.

Raw LC-MS spectral data were processed using Compound Discoverer (ver. 3.3, Thermo Fisher Scientific) for peak detection, retention time alignment, feature integration, and metabolite annotation. The resulting data set included metabolite names, accurate mass-to-charge ratios (*m*/*z*), retention times, peak areas, and annotation sources. Features without putative metabolite identification were excluded from further analysis. Metabolite annotations were supported by in-house spectral libraries, including mzCloud, mzVault, and MassList, which were validated using authentic chemical standards analyzed under identical chromatographic and mass spectrometric conditions.

Relative quantification was performed based on integrated peak areas from extracted ion chromatograms. Signal intensities were normalized to internal standards and bacterial culture optical density to account for analytical variability and differences in biomass prior to statistical analysis. Metabolite measurements were evaluated across biological replicates, and only features with consistent detection and acceptable analytical reproducibility were retained. Specifically, annotated metabolites with a coefficient of variation (CV) < 30% in pooled quality control (QC) samples were included. After the removal of duplicate annotations, the finalized metabolite list was used for downstream statistical analyses.

Univariate statistical analysis was conducted using Student’s *t*-test (*p* < 0.05) to identify significantly altered metabolites between gene knockout strains and the parent strain. Multivariate analyses, including principal component analysis (PCA) and hierarchical clustering, were performed to assess global metabolic shifts associated with gene deletions. Functional interpretation of significantly altered metabolites was carried out using KEGG pathway enrichment analysis implemented in MetaboAnalyst (version 6.0). Data visualization and additional statistical analyses were performed using MetaboAnalyst 6.0 and GraphPad Prism (GraphPad Software, San Diego, CA, USA).

## 3 Results

### 3.1 Overview of Metabolic Profiling in *E. coli* with TCA-Related Gene Deletions

Comprehensive untargeted metabolomics was performed to examine the metabolic consequences of single-gene knockouts in *E. coli* targeting the TCA cycle, TCA bypasses, and related pathways. Detailed information on metabolite coverage, analytical variability, and reproducibility across biological replicates was provided in Supplementary Figure 1. A total of 73 high-confidence metabolites were identified, spanning diverse compound classes including amino acids, nucleosides/nucleotides, carbohydrates, and organic acids (Supplementary Figure 1a). The distribution of CV values confirmed high reproducibility of the data set (Supplementary Figure 1b). PCA revealed clear clustering between the parent strain and the collection of TCA-related knockouts, indicating that single-gene deletions induce global metabolic shifts (Supplementary Figure 2a).

### 3.2 Core TCA Cycle Enzyme Knockouts Induce Distinct Metabolic Clusters

All reported metabolite changes represent relative abundance differences derived from normalized LC-MS peak intensities across biological replicates. Next, PCA was conducted to visualize the global metabolic differences between the parent strain and isolates carrying deletions in key TCA cycle enzymes. Knockouts of core TCA cycle genes resulted in the formation of two distinct metabolic clusters. Cluster 1, comprising mutants Δ*acnA*, Δ*fumA*, Δ*fumC*, Δ*mdh*, and Δ*icd*, displayed clear separation from the parent strain (Figure 1a). Within this group, mutants with closely related profiles, Δ*acnA*, Δ*fumA*, Δ*fumC*, and Δ*icd*, were defined as subcluster 1 (Figure 1b). PC1 accounted for 80.9% and 47.5% of the total variance in two independent analyses, respectively, indicating pronounced shifts in the metabolite composition relative to the parent. Cluster 2, including mutants Δ*acnB*, Δ*sucA*, Δ*sucB*, Δ*sucC*, Δ*sdhA*, Δ*sdhB*, Δ*sdhC*, and Δ*sdhD*, exhibited partial overlap with the parent strain, suggesting more moderate metabolic reprogramming (Figure 1d,e).

**Figure 1:**
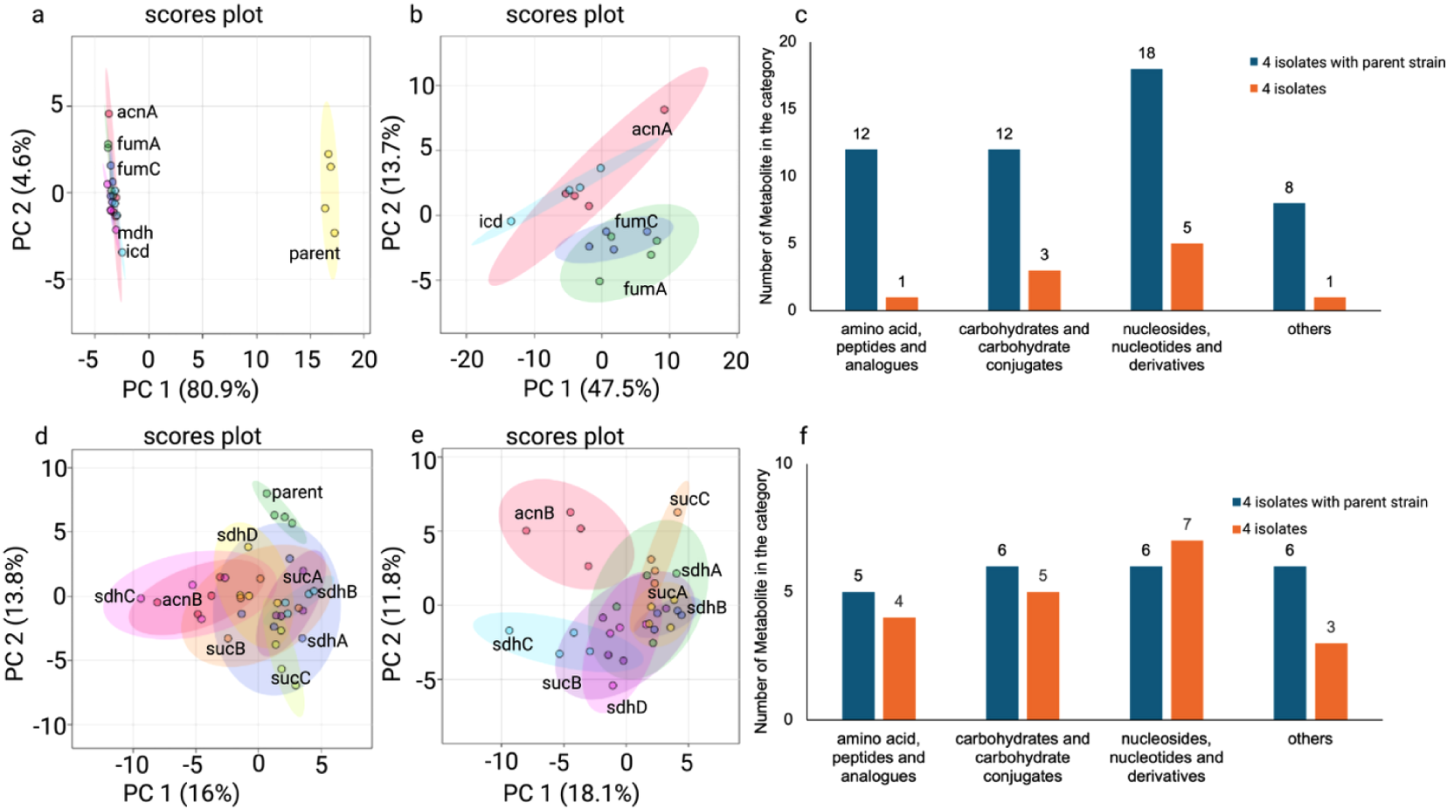
Comparative analysis of metabolic profiles between the parent strain and TCA core enzyme knockout strains, with subcluster characterization. (a) PCA plot comparing the parent strain with a distinct group of knockout strains (cluster 1). (b) PCA plot showing variation among knockout strains within cluster 1 (subcluster 1). (c) Distribution of compound classes for metabolites significantly altered in cluster 1, based on ANOVA (FDR-adjusted *p* < 0.05) and variable importance in projection (VIP) scores >1. (d) PCA plot comparing the parent strain with knockout strains grouped closely with it (cluster 2). (e) PCA plot showing intracluster variation among cluster 2 knockout strains, excluding the parent strain (subcluster 2). (f) Distribution of compound classes for significant metabolites in cluster 2 based on the same statistical criteria as in (c).

Metabolites showing significant differences between isolates and the parent strain, identified using FDR-adjusted *p*-values and a variable importance in projection (VIP) score >1, were classified into major biochemical categories. Such categories included amino acid, carbohydrate, and nucleotide-associated metabolites. In cluster 1, the greatest proportion of altered metabolites corresponded to nucleosides, nucleotides, and their derivatives (18 compounds), followed by amino acid and peptide analogues (12 compounds) and carbohydrate conjugates (12 compounds; Figure 1c). The number of significantly altered metabolites decreased markedly in subcluster 1. In contrast, cluster 2 displayed a more balanced distribution across categories, with 5 amino acid- and peptide-related metabolites, 6 carbohydrate conjugates, and 6 nucleoside- and nucleotide-related compounds (Figure 1f). Exclusion of the parent strain in subcluster 2 resulted in only minor changes in the number of metabolites within each category. A comprehensive comparison of metabolite intensity changes across cluster 1, subcluster 1, cluster 2, and subcluster 2 is presented in the heatmaps shown in Supplementary Figure 2b–f.

### 3.3 Isoform-Specific and Subunit-Specific Knockouts of TCA Enzymes Reveal Differential Reprogramming

Interestingly, the analysis of isoform-specific knockouts also demonstrated that deletion of aconitase isoforms (Δ*acnA* and Δ*acnB*) and fumarase isoforms (Δ*fumA* and Δ*fumC*) can produce distinct metabolic profiles (Figure 2). PCA indicated a greater divergence in Δ*acnB* compared to Δ*acnA* (Figure 2a), whereas Δ*fumA* and Δ*fumC* exhibited partial overlap compared with the parent strain (Figure 2c). Metabolite peak intensity comparisons highlighted class-specific perturbations in aconitase isoform comparisons (Figure 2b; Supplementary Figure 3). The peak intensity of glucose-6-phosphate (G6P), fructose lysine, lipoamide, and uridine-5′-triphosphate (UTP) was found to be significantly different between the parent strain and fumarase isoforms (Figure 2d). Comprehensive metabolite profiling revealed significant alterations in multiple pathways, including pyrimidine and purine metabolism, arginine biosynthesis, and alanine, aspartate, and glutamate metabolism, as illustrated in Supplementary Figure 4 a–d.

**Figure 2:**
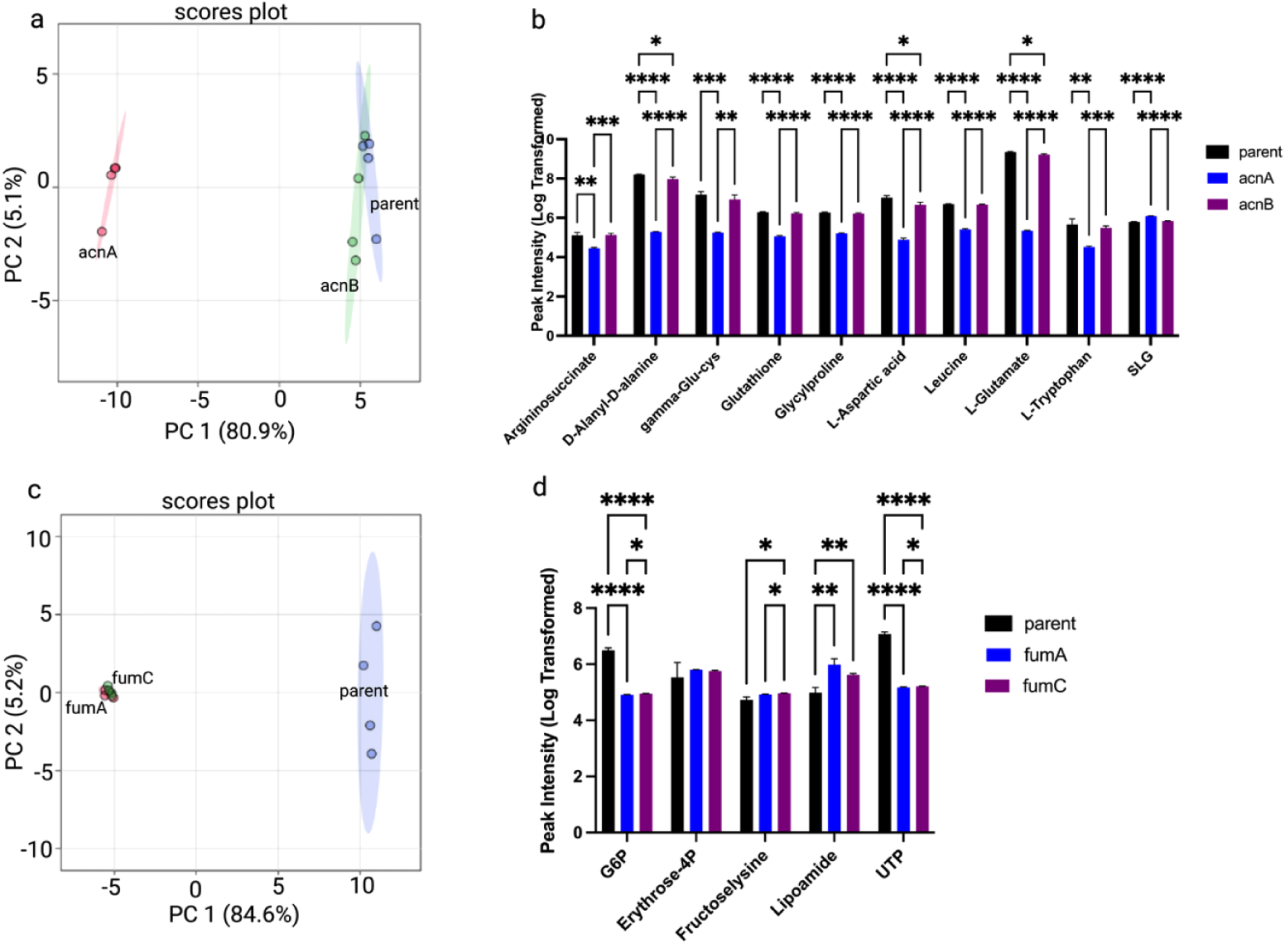
Impact of isoform-specific TCA enzyme knockouts on the metabolic profile of *E. coli*. (a) PCA score plot comparing the parent strain with knockout strains lacking Aconitate hydratase A (*acnA*) and Aconitate hydratase B (*acnB*). (b) Relative intensities of metabolites classified as amino acids, peptides, and analogues across the parent, Δ*acnA*, and Δ*acnB* strains. (c) PCA plot comparing the metabolic profiles of the parent strain with those of Fumarase A (*fumA*) and Fumarase C (*fumC*) knockout strains. (d) Differences in intensity for a subset of representative metabolites.

Knockouts of individual subunits of 2-oxoglutarate dehydrogenase (Δ*sucA* and Δ*sucB*) and succinate dehydrogenase (Δ*sdhA–D*) led to measurable and distinct metabolic alterations (Figure 3a–d). PCA revealed that Δ*sucA* and Δ*sucB* were partially separated from the parent strain (Figure 3a), and representative metabolites showed isoform- and subunit-dependent intensity changes (Figure 3b). Similarly, succinate dehydrogenase subunit knockouts produced heterogeneous but detectable shifts, suggesting that disruption of individual subunits induces localized metabolic perturbations rather than global rewiring (Figure 3c–d; Supplementary Figure 4 e-h). Despite changes in peak intensity, our data revealed changes in nicotinate and nicotinamide metabolism in Δ*sucA*/Δ*sucB* comparison (Supplementary Figure 4f) but none significantly altered pathway in Δ*sdhA–D* comparison (Supplementary Figure 4h).

**Figure 3:**
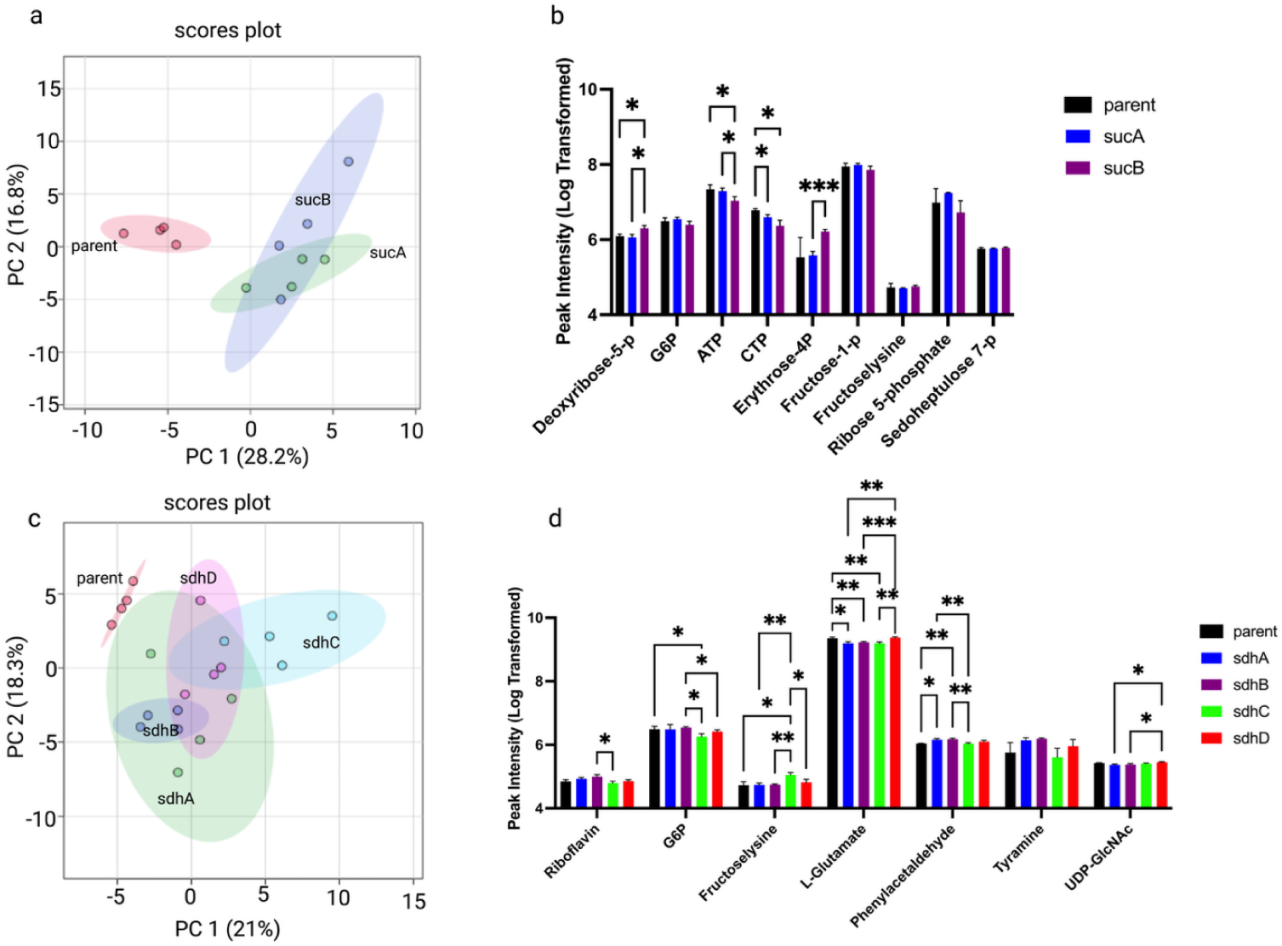
Effects of TCA enzyme subunit knockouts on the metabolic profile of *E. coli*. (a) PCA plot comparing the parent strain with knockout strains targeting subunits of 2-oxoglutarate dehydrogenase: the E1 component 1.2.4.2 **(Δ** *sucA*) and the other E1 component (Δ*sucB*). (b) Relative intensities of selected representative metabolites distinguishing the parent strain, Δ*sucA*, and Δ*sucB* strains. (c) PCA plot comparing the parent strain with knockout strains lacking subunits A, B, C, and D of succinate dehydrogenase (Δ*sdhA*, Δ*sdhB*, Δ*sdhC*, Δ*sdhD*). (d) Relative intensities of representative metabolites highlighting metabolic differences among the parent strain and the Δ*sdhA–D* knockout strains.

### 3.4 Knockouts of TCA-Associated and TCA Bypass Enzymes Impact Broader Metabolic Networks and Drive Pathway-Specific Metabolic Rewiring

Meanwhile, we observed that the deletion of malate dehydrogenase (Δ*mdh*) and malate:quinone oxidoreductase (Δ*mqo*), enzymes functionally linked to the TCA cycle, caused clear metabolic differences from the parent strain, as indicated in the PCA plot (Figure 4a). Both knockouts significantly affected pathways related to central carbon metabolism and redox balance, with representative metabolites spanning amino acids, nucleotides, and carbohydrate derivatives (Figure 4b–c; Supplementary Figure 5).

**Figure 4:**
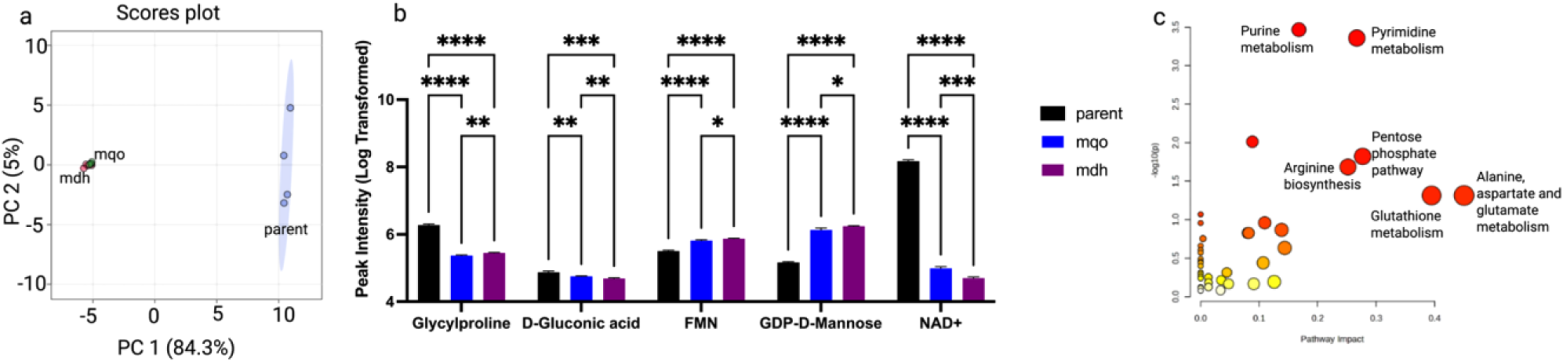
Effects of TCA-associated enzyme knockouts on the metabolic profile of *E. coli*. (a) PCA plot comparing the parent strain with knockout strains of malate dehydrogenase **(Δ** *mdh*) and malate:quinone oxidoreductase **(Δ** *mqo*). (b) Significantly altered metabolites between parent, Δ*mdh*, and Δ*mqo* strains. (c) Pathway analysis for Δ*mdh* and Δ*mqo*, based on significantly changed and representative metabolites.

Additionally, knockouts of glyoxylate bypass enzymes (Δ*aceA*, Δ*aceB*, and Δ*glcB*) and the malic enzyme (Δ*maeB*) revealed pronounced metabolic differences from the parent strain (Figure 5a–f). The metabolic profiles are not significantly among (Δ*aceA*, Δ*aceB*, Δ*glcB*), with only dUMP peak intensity statistically different between Δ*aceA* and Δ*glcB* (Figure 5c; Supplementary Figure 6a-b). Compared with the parent strain, Δ*maeB* predominantly altered carbohydrate and amino acid classes (Figure 5f; Supplementary Figure 6c-d). These results suggest that bypass pathways confer flexibility in the metabolite distribution and redox balance under gene disruptions.

**Figure 5:**
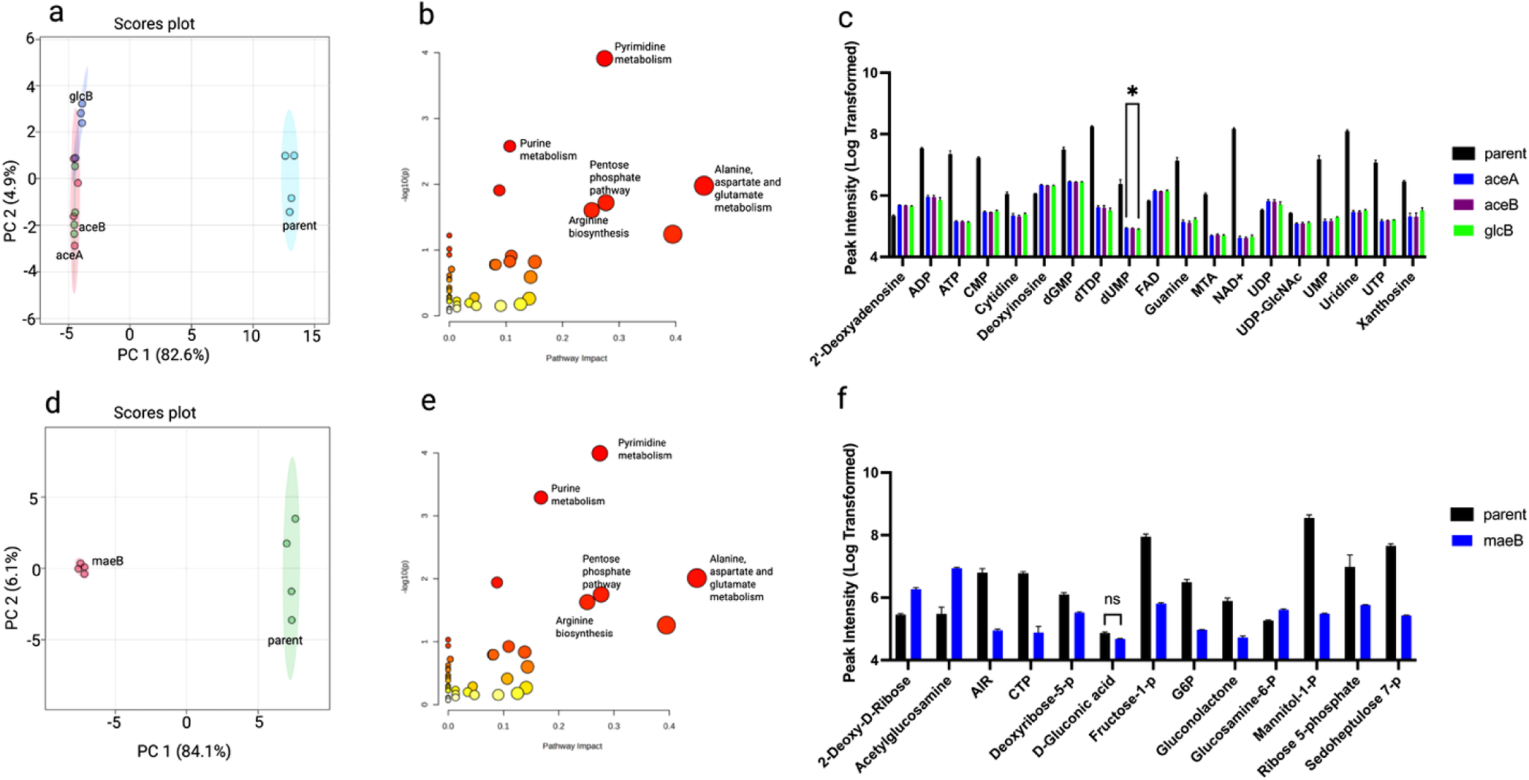
Metabolic and pathway-level effects of knocking out genes encoding TCA bypass enzymes in *E. coli*. (a–c) Glyoxylate bypass: (a) PCA plot comparing the parent strain with knockout strains Δ*aceA*, Δ*aceB*, and Δ*glcB*. (b) Pathway analysis based on significantly altered and representative metabolites in the glyoxylate bypass knockouts. (c) Differential abundance of metabolites within the nucleoside, nucleotide, and derivatives class. No isolate-wise significant metabolites except dUMP. (d–f) Substrate bypass via malic enzyme: (d) PCA plot comparing the parent strain with the Δ*maeB* knockout strain. (e) Pathway analysis based on significantly altered and representative metabolites in the substrate bypass knockouts Δ*maeB*. (f) Significantly altered metabolites classified as carbohydrates and carbohydrate conjugates. All metabolites are significantly different than d-gluconic acid.

### 3.5 Broader Effects on PPP, ETC, and Glycolysis/Gluconeogenesis

To further assess off-target and network-level effects, we analyzed knockouts of enzymes involved in PPP (8 mutants), ETC (16 mutants), and glycolysis/gluconeogenesis (9 mutants). PCA, pathway analysis, and heatmap visualization revealed that perturbations in these TCA-adjacent pathways propagated broadly through central metabolism, influencing multiple metabolite classes (Figure 6).

**Figure 6:**
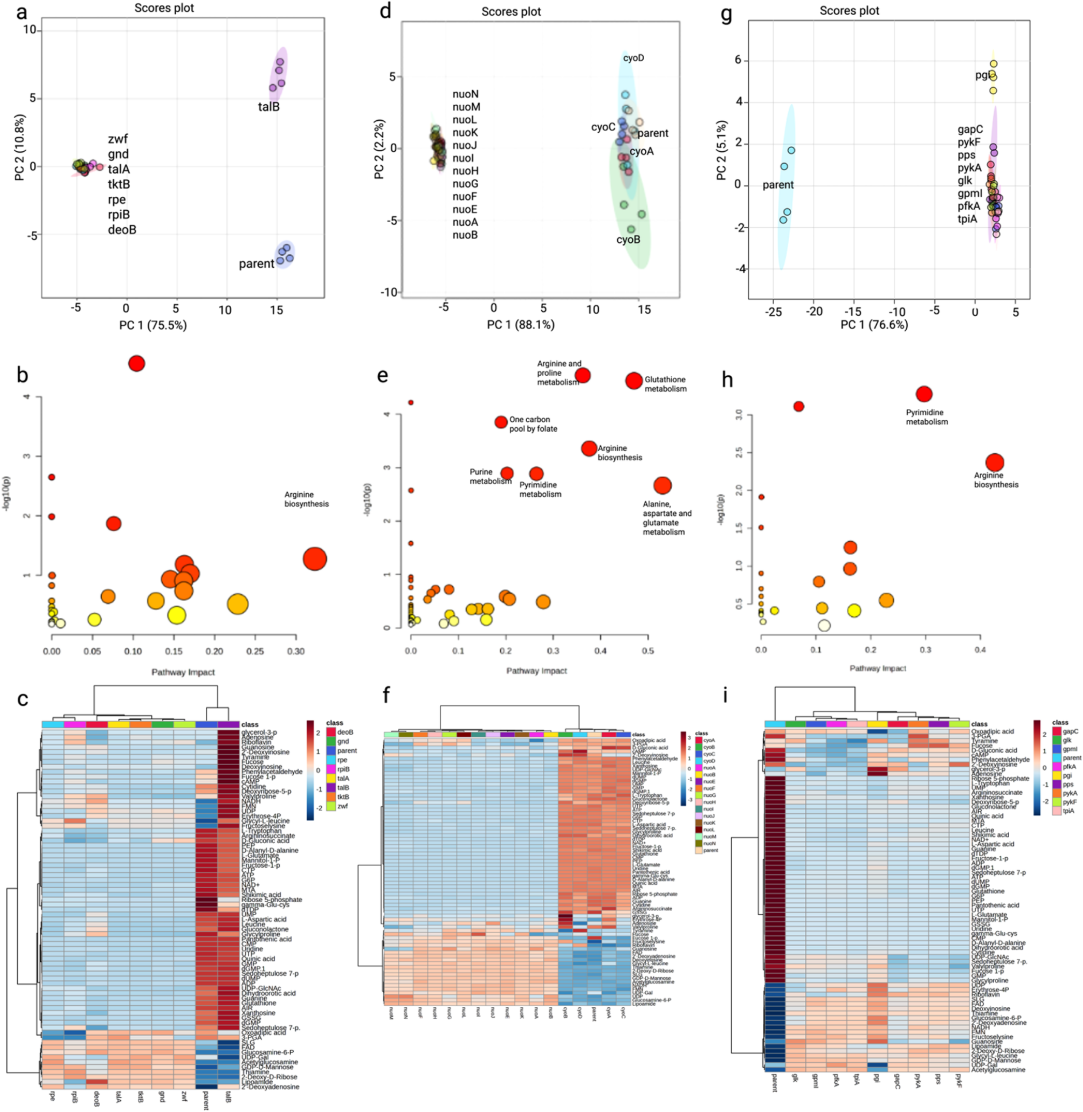
Metabolic and pathway-level effects of knocking out genes encoding enzymes functionally associated with the TCA cycle in *E. coli*. (a–c) Pentose Phosphate Pathway (PPP): (a) PCA plot comparing the parent strain and knockout strains with deletions in PPP-related genes. (b) Pathway enrichment analysis based on significantly altered and representative metabolites from the PPP knockouts. (c) Heatmap of metabolite intensities across the parent strain and PPP gene knockout strains. (d–f) Electron Transport Chain (ETC): (d) PCA plot of the parent strain and ETC-related gene knockout strains. (e) Pathway analysis reflecting ETC-related metabolic changes. (f) Heatmap showing metabolite intensity differences among the parent strain and ETC knockout strains. (g–i) Glycolysis and Gluconeogenesis: (g) PCA plot comparing the parent strain and gene knockouts involved in glycolysis/gluconeogenesis. (h) Pathway analysis based on significantly altered and representative metabolites. (i) Heatmap of metabolite intensities among parent and glycolysis/gluconeogenesis knockout strains.

Comprehensive metabolite profiling of PPP knockouts revealed significant alterations in nucleotide and sugar phosphate metabolism. Differentially abundant metabolites included purine and pyrimidine nucleotides such as adenosine triphosphate (ATP), adenosine diphosphate (ADP), uridine triphosphate (UTP), cytidine monophosphate (CMP), guanosine monophosphate (GMP), 2′-deoxyguanosine monophosphate (dGMP), and deoxyuridine monophosphate (dUMP). Alterations were also observed in sugar phosphate intermediates, including ribose-5-phosphate (R5P), glucose-6-phosphate (G6P), and sedoheptulose-7-phosphate (S7P).

ETC knockouts primarily affected redox-related metabolites. These included flavins such as riboflavin (vitamin B2), flavin adenine dinucleotide (FAD), and flavin mononucleotide (FMN), as well as nicotinamide cofactors, including nicotinamide adenine dinucleotide (NAD^+^) and its reduced form (NADH). Changes were also detected in thiol-related metabolites, including glutathione (GSH), oxidized glutathione (GSSG), and γ-glutamylcysteine (γ-Glu-Cys). Additional metabolites linked to cellular redox balance included pantothenic acid (vitamin B5), lipoamide, and S-lactoylglutathione (SLG).

Knockouts affecting glycolysis and gluconeogenesis resulted in broad shifts in carbohydrate and amino acid metabolism. Key carbohydrate intermediates showing altered abundance included G6P, phosphoenolpyruvate (PEP), R5P, S7P, and UDP-N-acetylglucosamine (UDP-GlcNAc). Amino acid-related changes were observed in l-aspartic acid (Asp), l-glutamic acid (Glu), and glutathione redox pairs (GSH/GSSG).

## 4 Discussion

In this study, untargeted metabolomics identified that the knockout of core TCA cycle enzymes in *E. coli* led to significant metabolic rewiring, demonstrating the network’s plasticity in maintaining metabolic function despite genetic perturbations. The clustering of knockout strains into distinct metabolic profiles (Figure 1) suggests that compensatory mechanisms are differentially activated, depending on the disrupted enzyme. Cluster 1 strains, which exhibited the most pronounced deviations from the wild type, likely rely on alternative pathways such as the glyoxylate shunt or malic enzyme bypass (Figure 5) to sustain carbon flux. In contrast, cluster 2 knockouts, which remained metabolically closer to the parent strain, may retain partial TCA functionality or utilize less disruptive bypass routes.

A plausible mechanistic explanation for the observed cluster-specific responses is that the engagement of bypass routes reflects the need to preserve key metabolic constraints following enzyme disruption, including carbon conservation, redox balance, and anaplerotic flux. Knockouts that disrupt decarboxylating steps or the generation of essential TCA intermediates may preferentially activate the glyoxylate shunt to minimize carbon loss while sustaining acetyl-CoA utilization,^5^ whereas perturbations that constrain oxidative flux or redox coupling may favor malic enzyme-mediated bypasses to rebalance cellular NAD­(P)H pools.^10^ Consistent with this framework, bypass pathway activation may be observed in Δ*acnA,* Δ*icd*, Δ*mdh*, Δ*fumA*, and Δ*fumC* mutants.

In contrast, knockouts that retain partial TCA functionality or effective regulatory compensation may rely on less disruptive rerouting, resulting in metabolite profiles closer to those of the parent strain. The observation that Δ*acnB*, Δ*sucA–C*, and Δ*sdhA–D* knockouts remain metabolically similar to the wild type likely reflects the buffering capacity of central metabolism under nutrient-rich conditions. Growth in LB medium reduces dependence on complete oxidative TCA cycling by providing abundant amino acids and alternative carbon sources, thereby enabling compensation through anaplerotic reactions and pathway redundancy.^11,12^ In addition, AcnB exhibits partial functional redundancy with AcnA under aerobic conditions,^13^ while perturbations of the 2-oxoglutarate dehydrogenase and succinate dehydrogenase complexes may preferentially affect respiratory coupling rather than carbon flux per se,^14,15^ limiting detectable changes in metabolite pool sizes. Because untargeted metabolomics captures steady-state metabolite abundances rather than flux, substantial metabolic rewiring may occur without pronounced accumulation or depletion of intermediates. Although direct flux measurements are required to quantitatively validate these mechanisms, the coordinated metabolite patterns observed across clusters support a model in which bypass pathway dominance represents an adaptive response to preserve metabolic homeostasis rather than a uniform rerouting of the central carbon metabolism.

The differential metabolic consequences observed for isoform-specific knockouts are consistent with established regulatory and stress-response mechanisms in *E. coli* (Figure 2). Aconitase isoforms acnA and acnB are differentially regulated by oxidative stress and iron availability, with acnA preferentially expressed under stress conditions and during the stationary phase,^16^ and acnB functioning primarily during aerobic exponential growth.^17^ In addition, the [4Fe–4S] cluster of AcnB is destabilized under oxidative stress or low iron conditions, leading to a loss of enzymatic activity.^13,18^ Disruption of acnA therefore likely compromises stress-adaptive capacity and redox sensing, resulting in more pronounced metabolic perturbations compared with acnB deletion. In contrast, deletion of fumA resulted in metabolic changes comparable to those observed for fumC. Although fumA encodes the primary fumarase under aerobic conditions and is sensitive to oxidative stress and iron limitation, whereas fumC is less abundant but more resistant to oxidative damage,^19^ the similar metabolic profiles suggest substantial functional buffering under the growth conditions used here. The absence of strong oxidative stress in the LB medium, potential engagement of bypass pathways, and compensatory regulation among fumarase isoforms may collectively limit the metabolic consequences of deleting either gene. These observations underscore that even ostensibly redundant isoenzymes can exhibit context-dependent functional overlap, complicating the prediction of metabolic outcomes following single-gene perturbations.

The differential metabolic consequences of subunit deletions in 2-oxoglutarate dehydrogenase (*sucA*, *sucB*) and succinate dehydrogenase (*sdhA–D*) (Figure 3) reveal the modularity of multienzyme complexes. *sdhC* and *sdhD* knockouts, which impair the quinone-binding subunits of succinate dehydrogenase, led to differential but pronounced succinate and succinylated arginine accumulation, consistent with their role in linking the TCA cycle to the ETC.^20^ These observations emphasize that not all subunits contribute equally to metabolic flux, with some playing more regulatory or structural roles than others. Direct detection of 2-oxoglutarate and its associated upstream and downstream metabolites would provide stronger evidence of the roles of suc subunits in modulating the conversion of 2-oxoglutarate to succinyl-CoA.

The activation of bypass routes, such as the glyoxylate shunt (*aceA*, *aceB*, *glcB*) and malic enzyme (*maeB*), in response to TCA cycle disruptions (Figure 5) illustrates *E. coli*’s metabolic flexibility. The glyoxylate shunt, which bypasses two decarboxylation steps of the TCA cycle, appears to be critical for maintaining carbon flux when succinate-generating enzymes (e.g., Δ*sdh* mutants) are impaired. This aligns with prior work showing that *aceA* and *aceB* are upregulated under carbon-limiting conditions.^21,22^ Meanwhile, the *maeB* knockout’s impact on carbohydrate metabolism (Figure 5f) suggests that the malic enzyme serves as a key anaplerotic node, replenishing pyruvate and supporting gluconeogenesis when the TCA cycle is compromised.

The broader metabolic network perturbations observed in PPP, ETC, and glycolysis/gluconeogenesis knockouts (Figure 6) highlight the interconnectedness of central metabolism. For instance, PPP disruptions altered nucleotide pools (Figure 6c), likely due to the reduced pentose precursors for nucleotide synthesis. Similarly, ETC knockouts affected redox-sensitive metabolites (Figure 6f), consistent with their role in maintaining the NAD+/NADH balance. The strong glycolytic/gluconeogenic shifts (Figure 6i) further suggest that carbon flux is dynamically rerouted in response to TCA cycle dysfunction, possibly to sustain ATP production via substrate-level phosphorylation.^23,24^ Figure 7 conceptually summarizes the cluster-specific metabolic rewiring observed in response to TCA-associated gene knockouts, highlighting bypass activation, buffering under LB conditions, and major metabolite class changes inferred from untargeted metabolomics.

**Figure 7:**
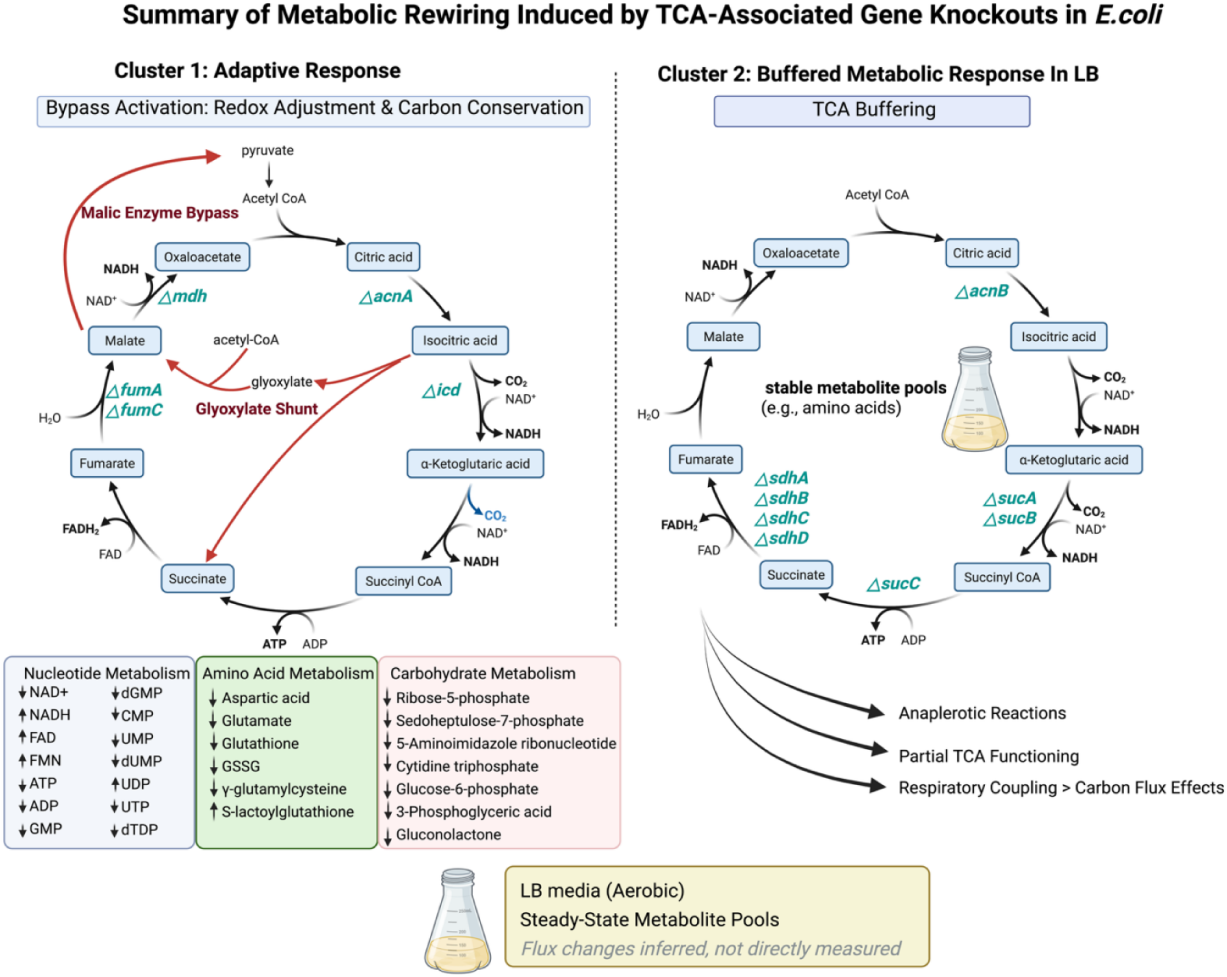
Summary schematic of metabolic rewiring induced by TCA-associated gene knockouts in *E. coli*. Untargeted metabolomics reveal two major metabolic response patterns. Cluster 1 knockouts exhibit adaptive responses characterized by the activation of bypass pathways and coordinated shifts in nucleotide, amino acid, and carbohydrate metabolism, whereas cluster 2 knockouts remain metabolically buffered under LB conditions. Arrows and annotations indicate an inferred pathway engagement based on steady-state metabolite changes rather than direct flux measurements.

Our findings have important implications for metabolic engineering and synthetic biology. First, they demonstrate that gene knockouts can have unpredictable off-target effects due to metabolic network flexibility. For example, attempts to increase succinate production by blocking the TCA cycle (e.g., Δ*sdh* knockouts) may inadvertently activate bypass routes, such as the glyoxylate shunt, thereby altering the intended product profile. Second, the strain-specific effects of isoform and subunit deletions suggest that precise pathway manipulation requires detailed knowledge of enzyme kinetics and regulatory interactions.

While this study provides a comprehensive view of metabolic responses to TCA cycle gene knockouts, several limitations remain that also highlight opportunities for future work. First, all experiments were conducted under aerobic conditions in LB medium; metabolic responses may differ substantially under alternative growth conditions such as minimal media or anaerobic environments. Second, although untargeted metabolomics enabled broad coverage of metabolic changes, it did not directly measure metabolic flux and was not optimal for detecting several key TCA cycle intermediates (e.g., citrate, 2-oxoglutarate, and succinyl-CoA). As a result, the metabolic rewiring and compensatory responses described here are inferred from steady-state metabolite abundance changes rather than quantitatively validated flux redistribution. Future studies integrating targeted metabolomics, stable isotope tracing, and flux analysis, ideally combined with complementary transcriptomic or proteomic measurements, will be essential to directly quantify pathway fluxes, validate inferred rewiring mechanisms, and uncover regulatory processes not captured in the present study.

## 5 Conclusions

This systematic untargeted metabolomics study demonstrates that single-gene knockouts in the *E. coli* central carbon metabolism trigger extensive and specific metabolic rewiring, revealing the network’s inherent plasticity. We identified two major clusters of core enzyme knockouts with distinct compensatory profiles and showed that isoform- and subunit-specific deletions produce differential metabolic outcomes, highlighting nuanced control beyond canonical pathways. The activation of bypass routes and significant off-target effects in glycolysis, PPP, and ETC underscores the deep interconnectivity of the central metabolism. This work establishes the power of untargeted metabolomics as a discovery tool to decipher complex gene-metabolite interactions and uncover hidden metabolic interlinks. The insights gained into the resilience and adaptability of *E. coli* metabolism provide a valuable framework for future efforts in functional genomics and metabolic engineering, where predicting and managing the systemic consequences of genetic modifications is paramount. Future studies integrating targeted flux analysis and multiomics approaches will be essential to fully quantify the rerouted metabolic flows and elucidate the underlying regulatory mechanisms that govern this remarkable network plasticity.

## Supplementary Material



## Data Availability

The raw metabolomics data can be accessed on Massive under study number MSV000099575 (ftp://MSV000099575@massive-ftp.ucsd.edu).

## Notes

Figure S1: Intrapolar metabolite coverage and analytical variability in *E. coli*. Figure S2: PCA and heatmap analyses of metabolite profiles across TCA-related gene knockout strains and the parent strain. Figure S3: Differential metabolite intensities among TCA enzyme isoform knockout strains. Figure S4: Heatmap-based metabolite profiling and pathway-level alterations associated with TCA enzyme isoform or subunit knockouts. Figure S5: Metabolic alterations associated with knockouts of enzymes functionally linked to the TCA cycle. Figure S6: Metabolite intensity changes in TCA bypass enzyme knockout strains across major compound classes (PDF)

## Abbreviations

3-PGA: 3-phosphoglyceric acid
ADP: adenosine diphosphate
AIR: 5-Aminoimidazole ribonucleotide
Argininosuccinate: argininosuccinic acid
ATP: adenosine triphosphate
Acetylglucosamine: N-acetyl-beta-d-glucosamine
CMP: cytidine monophosphate
CTP: cytidine triphosphate
dGMP: deoxyguanosine monophosphate
Deoxyribose-5-P: 2-deoxy-d-ribose 5-phosphate
dTDP: deoxythymidine diphosphate
dUMP: deoxyuridine monophosphate
Erythrose-4P: d-Erythrose 4-phosphate
FAD: flavin adenine dinucleotide
FMN: flavin mononucleotide
Fucose-1-P: l-fucopyranose 1-phosphate
Fructose-1-P: d-fructose 1-phosphate
GDP-D-Mannose: guanosine diphosphate mannose
G6P: glucose-6-phosphate
Gluconolactone: d-glucono-delta-lactone
Glycerol-3-P: glycerol 3-phosphate
GMP: guanosine monophosphate
gamma-Glu-Cys: gamma-l-glutamyl-l-cysteine
l-Aspartic acid: l-(+)-Aspartic acid
Mannitol-1-P: d-mannitol 1-phosphate
MTA: adenylthiomethylpentose
NAD+: nicotinamide adenine dinucleotide (NAD+)
NADH: nicotinamide adenine dinucleotide, reduced form
PEP: phosphoenolpyruvic acid
Ribose-5-P: ribose 5-phosphate
Sedoheptulose-7-P: d-sedoheptulose 7-phosphate
SLG: s-lactoylglutathione
UDP: uridine diphosphate
UDP-GlcNAc: uridine diphosphate N-acetylglucosamine
UDP-Gal: uridine diphosphate galactose
UTP: uridine-5′-triphosphate
UMP: uridine monophosphate
