# Supplementary material for: Dissecting Metabolic Rewiring and Gene-Metabolite Interactions by Utilizing Untargeted Metabolomics and Single-Gene Knockouts in the Model Microorganism E. coli
Source: J Am Soc Mass Spectrom. 2026 Mar 16;37(4):967–76. doi: 10.1021/jasms.5c00454 (PMC13047672; doi:10.1021/jasms.5c00454)
Supplement: Supplementary file 1 [file js5c00454_si_001.pdf]

Supporting information for

Dissecting Metabolic Rewiring and Gene-Metabolite Interactions by Utilizing Untargeted Metabolomics and Single Gene Knockouts in Model Microorganism E. coli

Xinru Pang, Li Chen, Huan Zhang, Shiqi Zhang, Jiangjiang Zhu*

Department of Human Sciences & James Comprehensive Cancer Center, The Ohio State University, Columbus, OH 43210, United States of America

Corresponding author:

Jiangjiang Zhu, PhD

Zhu.2484@osu.edu

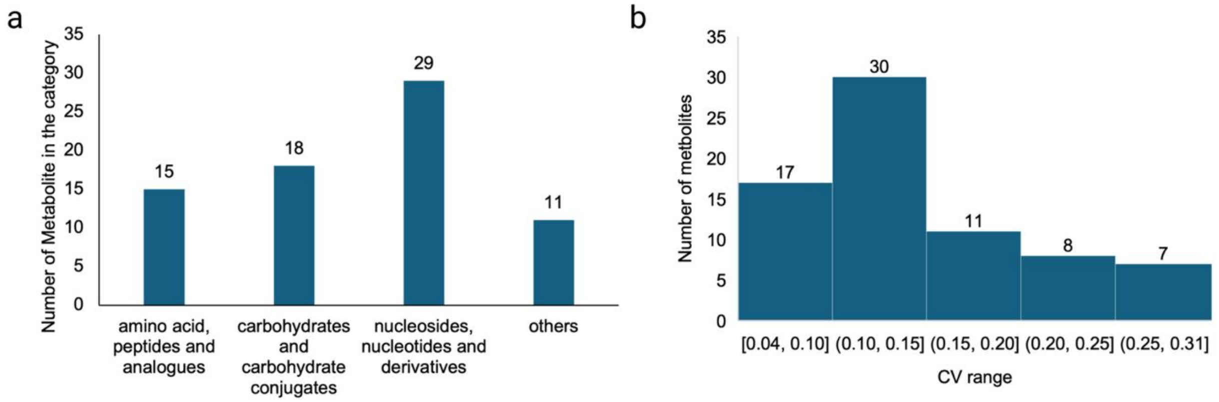

Supplementary Figure 1. Overview of the intrapolar metabolite profile in *E. coli***.**

(a) Number of detected metabolites categorized by compound class. (b) Distribution of the coefficient of variation (CV) across 73 metabolites associated with *E. coli* central metabolism.

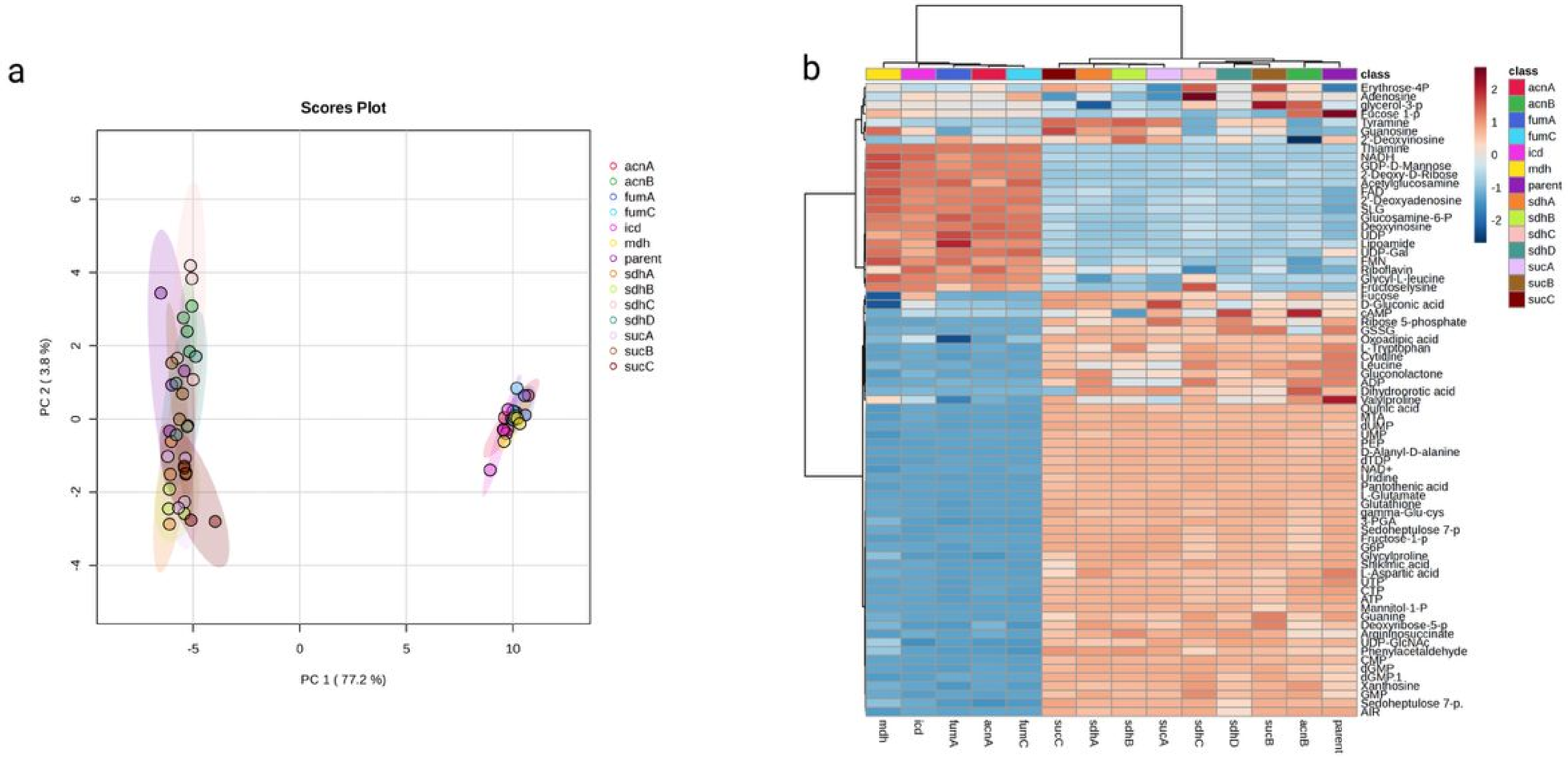
a
Scores Plot
PC 1 (77.2 %)
PC 2 (3.8 %)
acnA
acnB
fumA
fumC
icd
mdh
parent
sdhA
sdhB
sdhC
sdhD
sucA
sucB
sucC
b
class
Erythrose-4P
Adenosine
glycerol-3-p
Fucose 1-p
Tyramine
Guanosine
2-Deoxyinosine
Thiamine
NADH
GDP-D-Mannose
2-Deoxy-D-Ribose
Acetylglucosamine
FAD
2-Deoxyadenosine
SLG
Glucosamine-6-P
Deoxyinosine
UDP
Lipoamide
UDP-Gal
FMN
Riboflavin
Glycyl-L-leucine
Fructoselysine
Fucose
cAMP
D-Gluconic acid
Ribose 5-phosphate
GSSG
Oxoadipic acid
L-Tryptophan
Cytidine
Leucine
Gluconolactone
ADP
Dihydroorotic acid
Valylproline
Quinic acid
MTA
dUMP
UMP
PEP
D-Alanyl-D-alanine
dTDP
NAD+
Uridine
Pantothenic acid
L-Glutamate
Glutathione
gamma-Glu-cys
3-PGA
Sedoheptulose 7-p
Fructose-1-p
G6P
Glycylproline
Shikimic acid
L-Aspartic acid
UTP
CTP
ATP
Mannitol-1-P
Guanine
Deoxyribose-5-p
Argininosuccinate
UDP-GlcNAc
Phenylacetaldehyde
CMP
dGMP
dGMP.1
Xanthosine
GMP
Sedoheptulose 7-p.
AIR
mdh
icd
fumA
acnA
fumC
sucC
sdhA
sdhB
sucA
sdhC
sdhD
sucB
acnB
parent

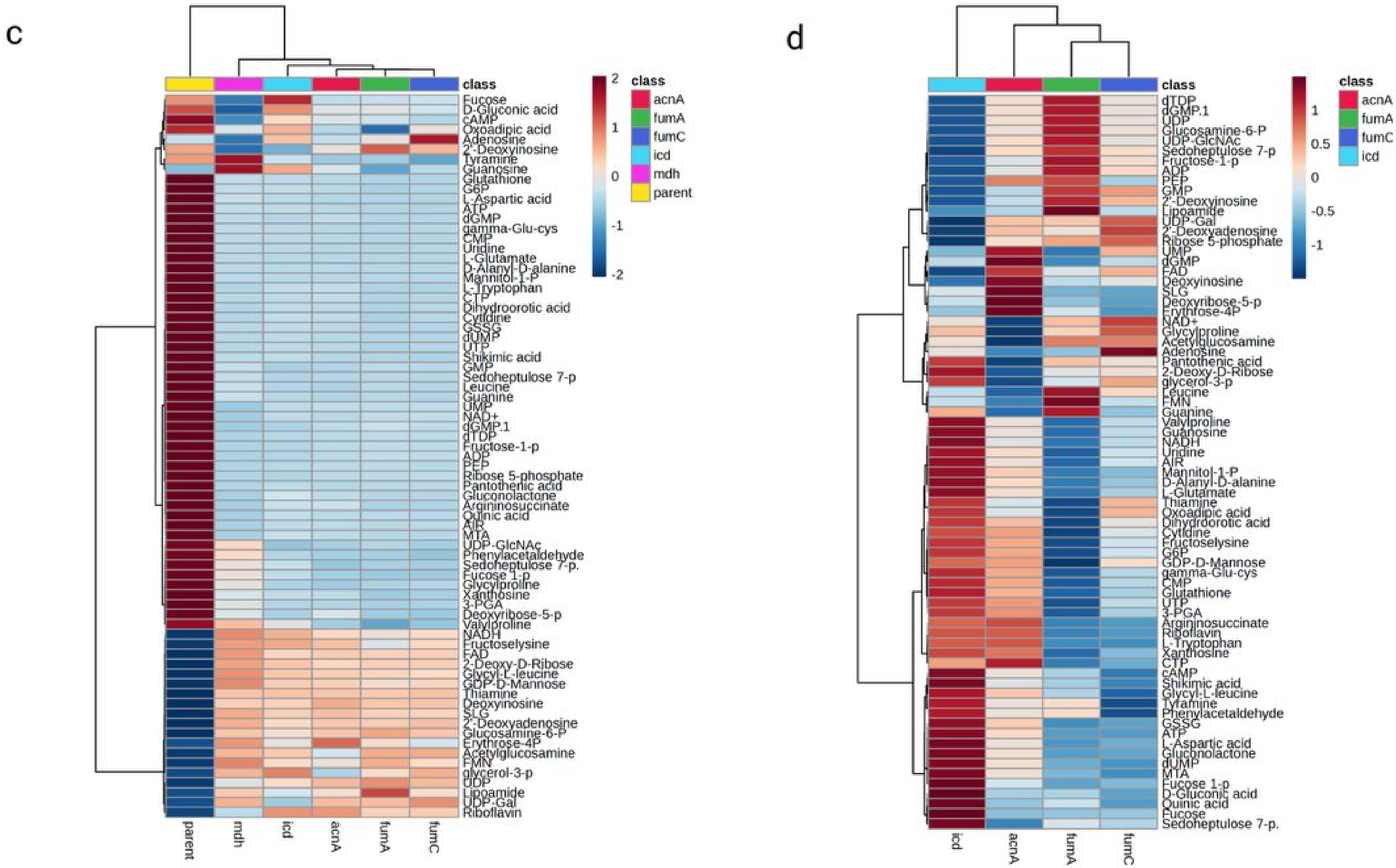
c
class
Fucose
D-Gluconic acid
cAMP
Oxoadipic acid
Adenosine
2'-Deoxyinosine
Tyramine
Guanosine
Glutathione
G6P
L-Aspartic acid
ATP
dGMP
gamma-Glu-cys
CMP
Uridine
L-Glutamate
D-Alanyl-D-alanine
Mannitol-1-P
L-Tryptophan
CTP
Dihydroorotic acid
Cytidine
GSSG
dUMP
UTP
Shikimic acid
GMP
Sedoheptulose 7-p
Leucine
Guanine
UMP
NAD+
dGMP.1
dTDP
Fructose-1-p
ADP
PEP
Ribose 5-phosphate
Pantothenic acid
Gluconolactone
Argininosuccinate
Quinic acid
AIR
MTA
UDP-GlcNAc
Phenylacetaldehyde
Sedoheptulose 7-p.
Fucose 1-p
Glycylproline
Xanthosine
3-PGA
Deoxyribose-5-p
Valylproline
NADH
Fructoselysine
FAD
2-Deoxy-D-Ribose
Glycyl-L-leucine
GDP-D-Mannose
Thiamine
Deoxyinosine
SLG
2'-Deoxyadenosine
Glucosamine-6-P
Erythrose-4P
Acetylglucosamine
FMN
glycerol-3-p
UDP
Lipoamide
UDP-Gal
Riboflavin
parent
mdh
icd
acnA
fumA
fumC
2
1
0
-1
-2
class
acnA
fumA
fumC
icd
mdh
parent
d
class
dTDP
dGMP.1
UDP
Glucosamine-6-P
UDP-GlcNAc
Sedoheptulose 7-p
Fructose-1-p
ADP
PEP
GMP
2'-Deoxyinosine
Lipoamide
UDP-Gal
2'-Deoxyadenosine
Ribose 5-phosphate
UMP
dGMP
FAD
Deoxyinosine
SLG
Deoxyribose-5-p
Erythrose-4P
NAD+
Glycylproline
Acetylglucosamine
Adenosine
Pantothenic acid
2-Deoxy-D-Ribose
glycerol-3-p
Leucine
FMN
Guanine
Valylproline
Guanosine
NADH
Uridine
AIR
Mannitol-1-P
D-Alanyl-D-alanine
L-Glutamate
Thiamine
Oxoadipic acid
Dihydroorotic acid
Cytidine
Fructoselysine
G6P
GDP-D-Mannose
gamma-Glu-cys
CMP
Glutathione
UTP
3-PGA
Argininosuccinate
Riboflavin
L-Tryptophan
Xanthosine
CTP
cAMP
Shikimic acid
Glycyl-L-leucine
Tyramine
Phenylacetaldehyde
GSSG
ATP
L-Aspartic acid
Gluconolactone
dUMP
MTA
Fucose 1-p
D-Gluconic acid
Quinic acid
Fucose
Sedoheptulose 7-p.
icd
acnA
fumA
fumC
1
0.5
0
-0.5
-1
class
acnA
fumA
fumC
icd

e

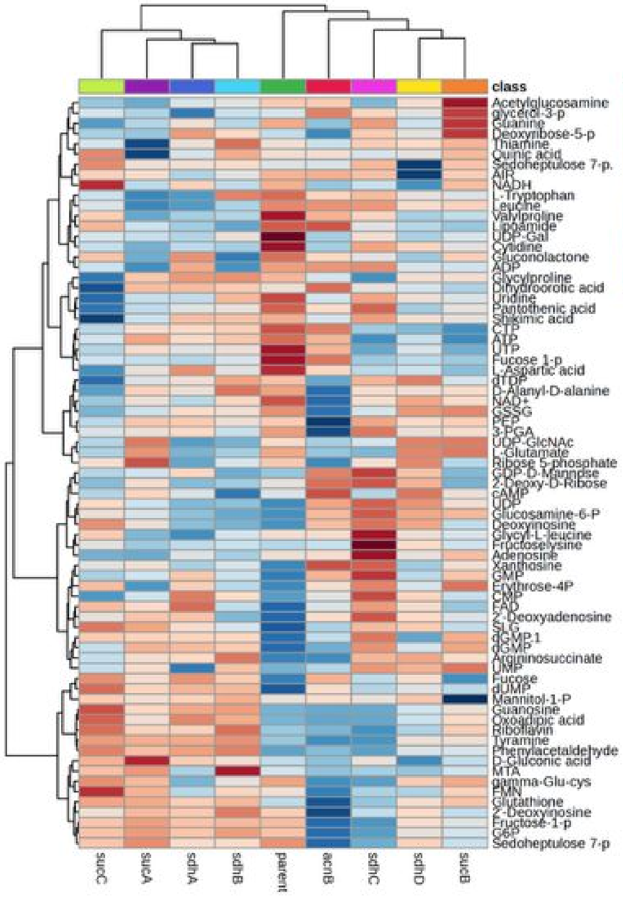
class
acnB
parent
sdhA
sdhB
sdhC
sdhD
sucA
sucB
sucC
2
1
0
-1
-2
sucC
sucA
sdhA
sdhB
parent
acnB
sdhC
sdhD
sucB

f

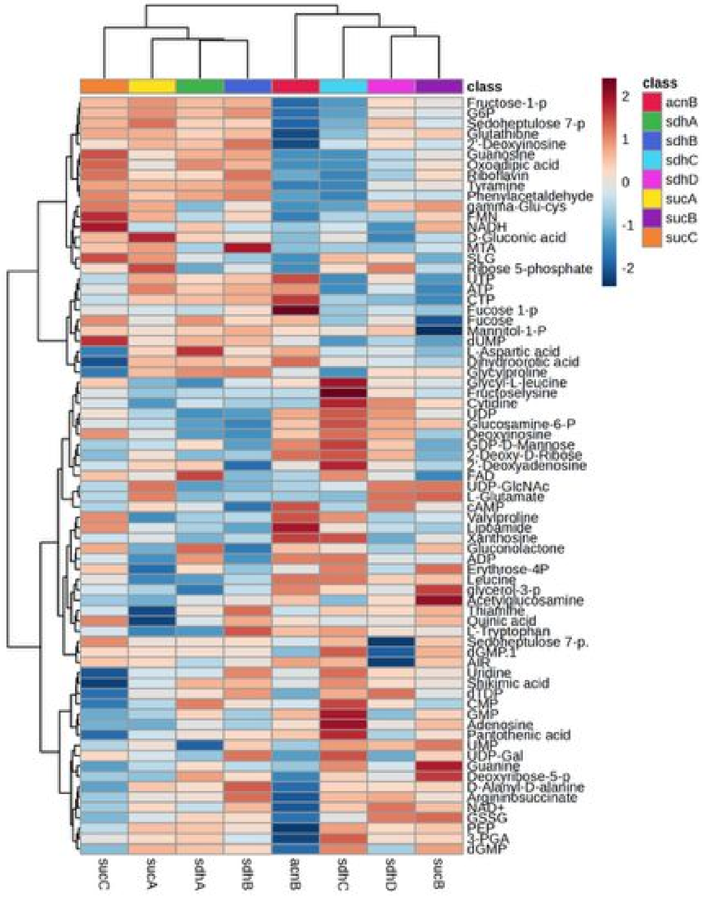
class
acnB
sdhA
sdhB
sdhC
sdhD
sucA
sucB
sucC
2
1
0
-1
-2
sucC
sucA
sdhA
sdhB
acnB
sdhC
sdhD
sucB

Supplementary Figure 2. Comparative analysis of metabolite profiles between *E. coli* TCA-related gene knockout strains and the parent strain.

(a) PCA plot showing clustering patterns of the parent strain and knockout strains targeting TCA-related enzymes. (b) Heatmap of metabolite profiles across all TCA-related gene knockout strains and the parent strain. (c) Heatmap highlighting metabolite differences between Cluster 1 knockout strains and the parent strain. (d) Heatmap displaying internal variation among Cluster 1 knockout strains, excluding the parent strain (subcluster 1). (e) Heatmap showing metabolic similarities between Cluster 2 knockout strains and the parent strain. (f) Heatmap of Cluster 2 knockout strains excluding the parent strain (subcluster 2).

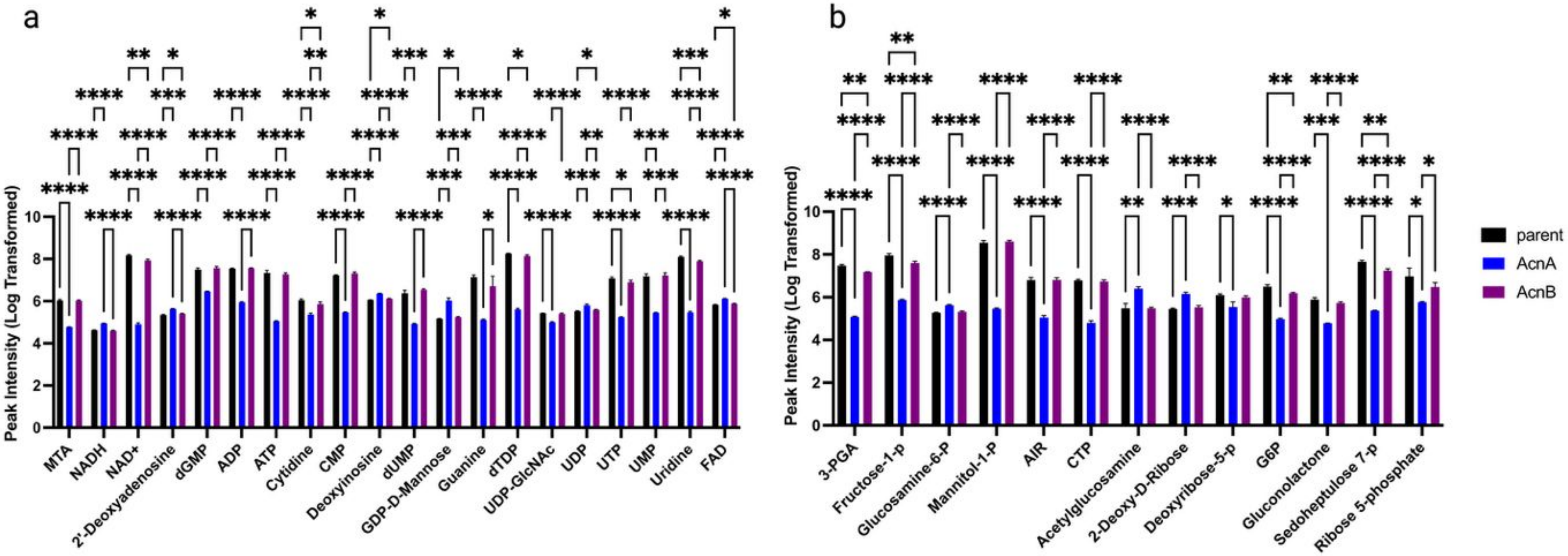

Supplementary Figure 3. Metabolite intensity differences among the parent strain, Δ*acnA*, and Δ*acnB* knockout strains**.** (a) Relative intensities of metabolites classified as nucleosides, nucleotides, and derivatives, comparing the parent strain with Δ*acnA* and Δ*acnB* isolates. (b) Relative intensities of metabolites classified as carbohydrates and carbohydrate conjugates across the same strains

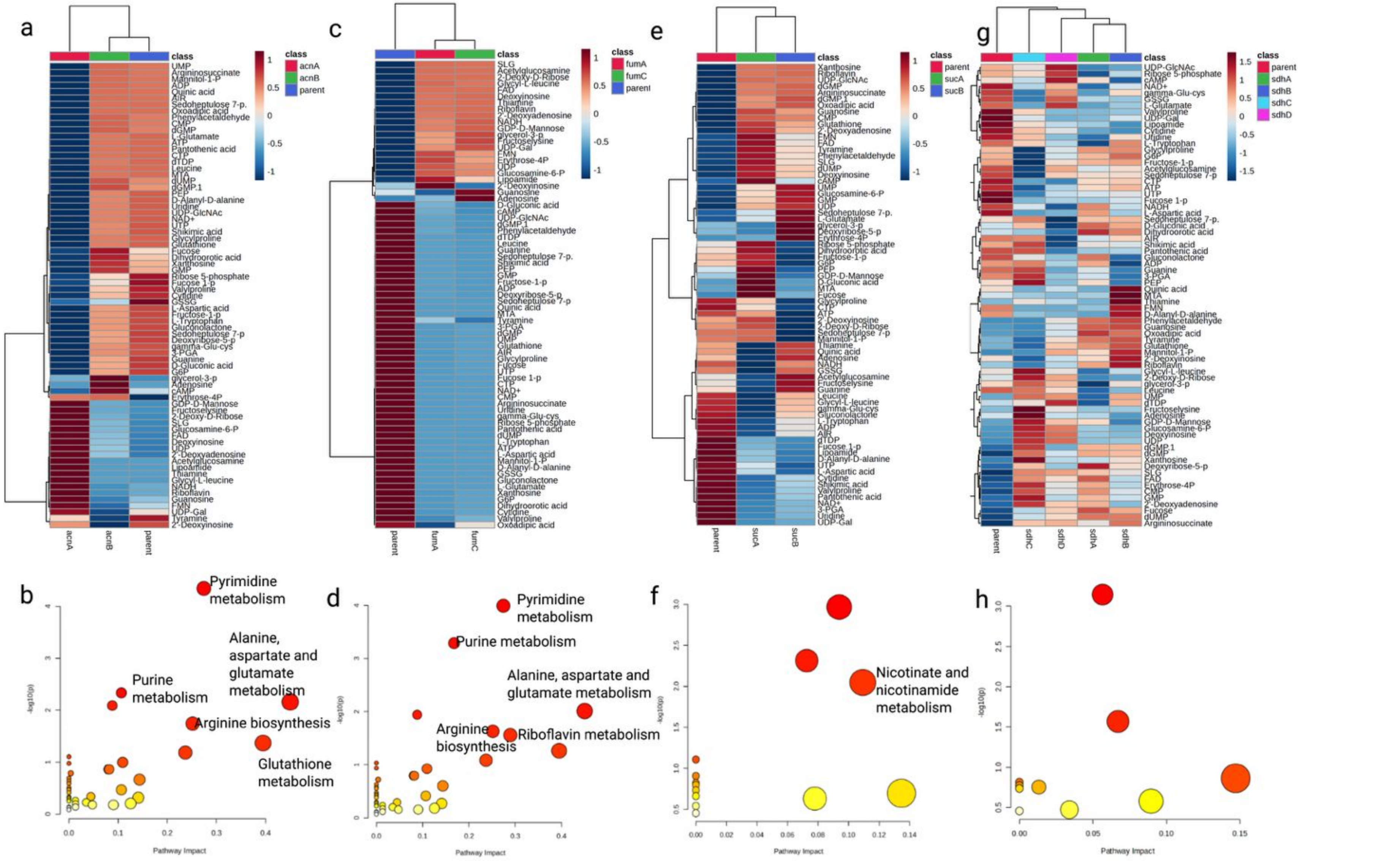
a
c
e
g
b
Pyrimidine metabolism
Alanine, aspartate and glutamate metabolism
Purine metabolism
Arginine biosynthesis
Glutathione metabolism
d
Pyrimidine metabolism
Purine metabolism
Alanine, aspartate and glutamate metabolism
Arginine biosynthesis
Riboflavin metabolism
f
Nicotinate and nicotinamide metabolism
h
Pathway Impact

Supplementary Figure 4. Metabolite intensity changes and pathway-level alterations in TCA enzyme knockout strains.

(a) Heatmap comparing metabolite intensities among the parent strain, Δ*acnA*, and Δ*acnB* strains. (b) Pathway analysis for Δ*acnA* and Δ*acnB*, based on significantly altered and representative metabolites. (c–d) Heatmap and corresponding pathway analysis for the parent strain, Δ*fumA*, and Δ*fumC* strains. (e–f) Heatmap and pathway analysis comparing the parent strain with Δ*sucA* and Δ*sucB* strains. (g–h) Heatmap and pathway analysis comparing the parent strain with succinate dehydrogenase subunit knockout strains (Δ*sdhA*, Δ*sdhB*, Δ*sdhC*, Δ*sdhD*).

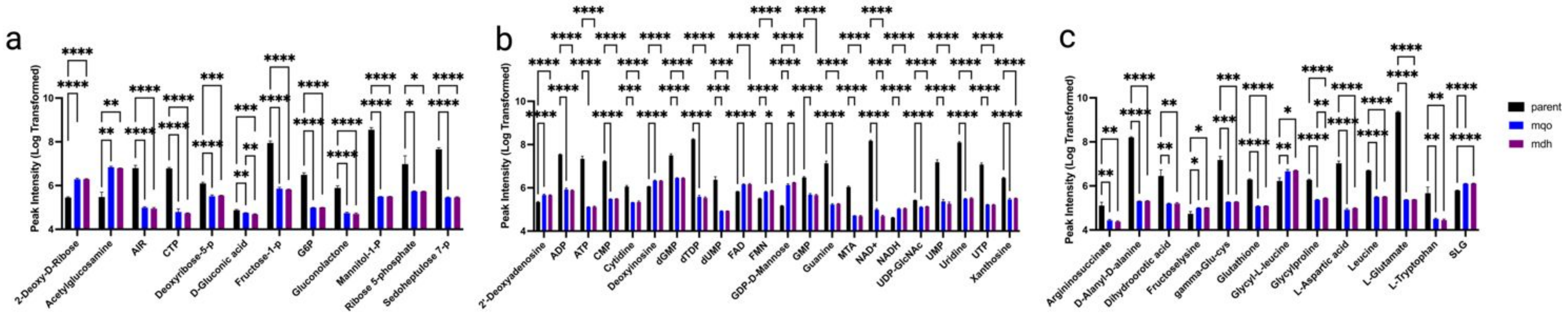

Supplementary Figure 5. Metabolite intensity differences among the parent strain, Δ*mqo*, and Δ*mdh* knockout strains across distinct compound classes.

(a) Relative intensities of metabolites classified as **c**arbohydrates and carbohydrate conjugates. (b) Relative intensities of metabolites in the nucleosides, nucleotides, and derivatives class. (c) Relative intensities of metabolites belonging to the amino acids, peptides, and analogues class**.**

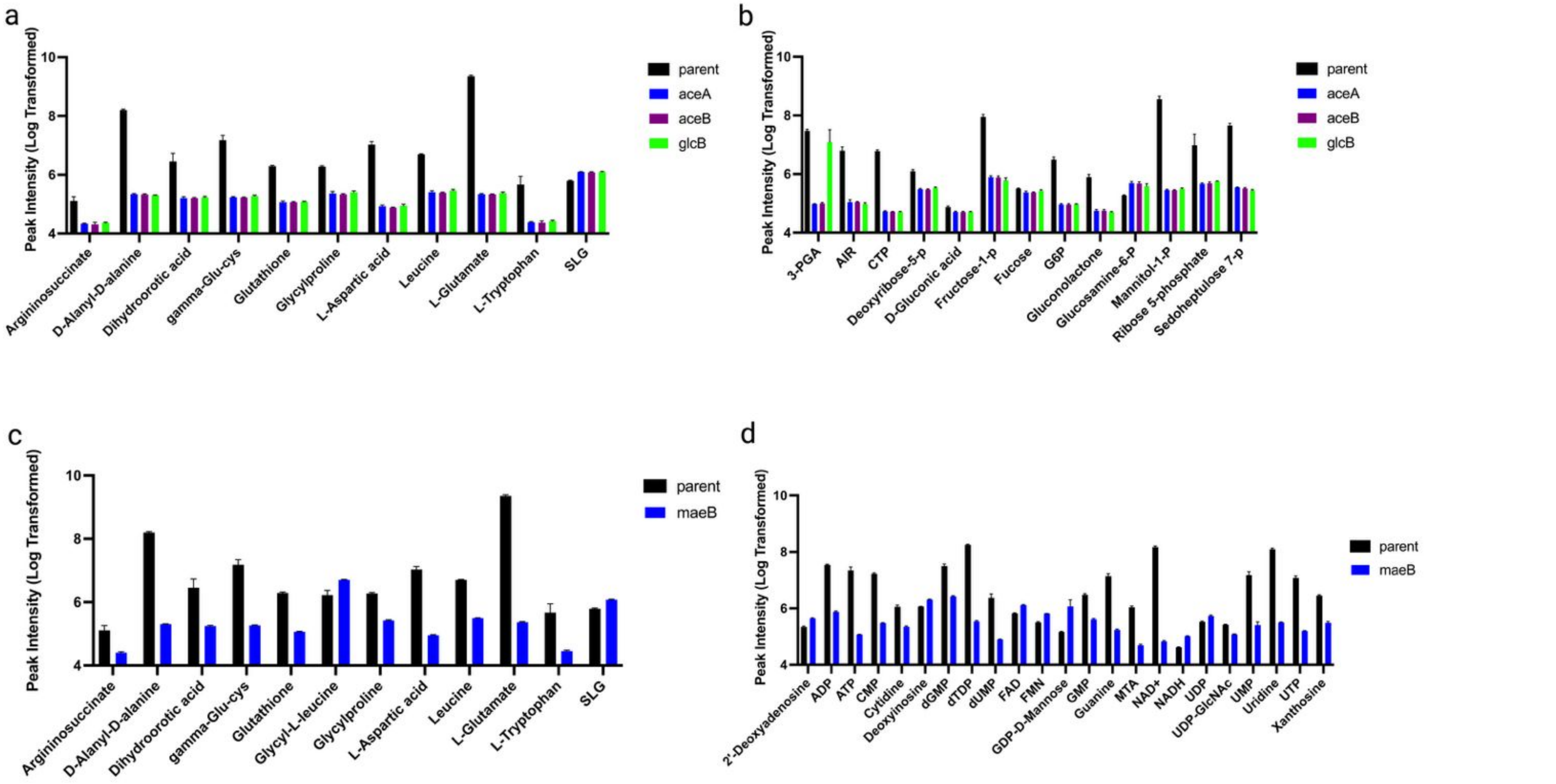
a
Peak Intensity (Log Transformed)
10
8
6
4
parent
aceA
aceB
glcB
Argininosuccinate
D-Alanyl-D-alanine
Dihydroorotic acid
gamma-Glu-cys
Glutathione
Glycylproline
L-Aspartic acid
Leucine
L-Glutamate
L-Tryptophan
SLG
b
Peak Intensity (Log Transformed)
10
8
6
4
parent
aceA
aceB
glcB
3-PGA
AIR
CTP
Deoxyribose-5-p
D-Gluconic acid
Fructose-1-p
Fucose
G6P
Gluconolactone
Glucosamine-6-P
Mannitol-1-P
Ribose 5-phosphate
Sedoheptulose 7-p
c
Peak Intensity (Log Transformed)
10
8
6
4
parent
maeB
Argininosuccinate
D-Alanyl-D-alanine
Dihydroorotic acid
gamma-Glu-cys
Glutathione
Glycyl-L-leucine
Glycylproline
L-Aspartic acid
Leucine
L-Glutamate
L-Tryptophan
SLG
d
Peak Intensity (Log Transformed)
10
8
6
4
parent
maeB
2'-Deoxyadenosine
ADP
ATP
CMP
Cytidine
Deoxyinosine
dGMP
dTDP
dUMP
FAD
FMN
GDP-D-Mannose
GMP
Guanine
MTA
NAD+
NADH
UDP
UDP-GlcNAc
UMP
Uridine
UTP
Xanthosine

Supplementary Figure 6. Metabolite intensity changes in TCA bypass enzyme knockout strains across compound classes.

(a–b) Glyoxylate bypass:

(a) Relative intensities of metabolites classified as amino acids, peptides, and analogues in the parent strain versus Δ*aceA*, Δ*aceB*, and Δ*glcB* knockout strains.

(b) Relative intensities of carbohydrates and carbohydrate conjugates comparing the same strains.

(c–d) Substrate bypass via malic enzyme:

(c) Comparison of metabolite intensities in the amino acids, peptides, and analogues class between the parent strain and Δ*maeB*.

(d) Relative intensities of nucleosides, nucleotides, and derivatives in Δ*maeB* versus the parent strain.
